# Edema Bullae Mimicking Disseminated Herpes Zoster

**DOI:** 10.7759/cureus.1780

**Published:** 2017-10-17

**Authors:** Stella X Chen, Philip R Cohen

**Affiliations:** 1 School of Medicine, University of California, San Diego; 2 Department of Dermatology, University of California, San Diego

**Keywords:** acute, bullae, dermatomal, disseminated herpes zoster, edema, nondermatomal disseminated herpes zoster, varicella, virus, zoster, zosteriform

## Abstract

Edema bullae typically forms at the site of skin swelling during acute states of volume overload, most commonly during renal or cardiac failure. Herpes zoster is a reactivation of latent varicella zoster virus that typically presents as painful vesicles in a dermatomal distribution. In immunocompromised individuals, disseminated herpes zoster skin manifestations may occur with several lesions in multiple dermatomes or widespread individual lesions or both, even visceral organs can be involved. Additionally, many conditions are known to mimic the lesions and distribution of herpes zoster. A 53-year-old immunosuppressed male with a history of renal transplant presented with dermatomal and non-dermatomal, disseminated herpes zoster that was confirmed by polymerase chain reaction testing. After one week of intravenous antiviral therapy during which his virus infection-associated lesions were resolved, new blisters developed near the insertion site of a peripheral venous line located on a previously uninvolved yet swollen upper extremity. The varicella zoster virus disease was initially suspected, but lab studies and skin biopsy of a blister excluded progressive or persistent viral infection and established a diagnosis of acute edema bullae. The blisters resolved following removal of the peripheral catheter. Acute edema bullae should be added to the list of mimickers of disseminated varicella zoster virus infection.

## Introduction

Edema bullae are the blisters that occur following the swelling at an affected site, most commonly in the lower extremities of the patients with acute exacerbation of fluid overload or chronic leg swelling [1]. Herpes zoster, also known as shingles is a viral infection resulting from the varicella zoster virus (VZV) reactivation and presents as painful blisters in a dermatomal distribution. Rarely, disseminated herpes zoster may occur in immunocompromised patients in which the skin manifestations involve multiple dermatomes or widespread individual lesions or both. Other conditions that have been reported to mimic zosteriform lesions include Staphylococcal cutaneous infection [2], herpes simplex virus [3], cutaneous metastases [4], and even eosinophilic dermatosis of the hematologic malignancy [5]. Here, we report a case of edema blisters masquerading as herpes zoster in an immunosuppressed male with resolving disseminated herpes zoster infection.

## Case presentation

A 53-year-old immunosuppressed male with the history of the kidney transplant presented with two weeks of painful vesicles on his left forearm, left leg, and the abdomen. His past medical history was significant for kidney transplant four years prior and disseminated herpes zoster two years prior. His daily medications included 7.5 milligrams of prednisone, 6 milligrams of Tacrolimus, and 720 milligrams of mycophenolate sodium.

The cutaneous examination showed individual and grouped, erythematous-based vesicles located in a dermatomal distribution on the left side of his abdomen, and more than 20 individual lesions in a diffuse, nondermatomal distribution on his body. Initial polymerase chain reaction testing of a lesional swab was positive for varicella zoster virus and negative for herpes simplex virus. He was started on 10 mg/kg of intravenous acyclovir three times per day for disseminated herpes zoster involving multiple dermatomes but without organ or central nervous system involvement.

**Figure 1:**
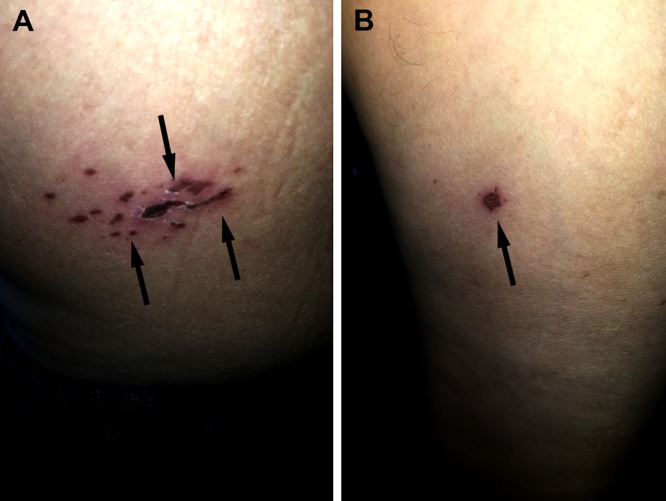
Resolving lesions of the disseminated varicella zoster virus in an immunosuppressed 53-year-old male with a history of the kidney transplant. Eschars at the sites of disseminated herpes zoster (arrows) located in a dermatomal distribution of the thoracic 10 (T10) and thoracic 11 (T11) on the left lower abdomen (A) and as a single lesion on the left upper anterior thigh (B).

**Figure 2:**
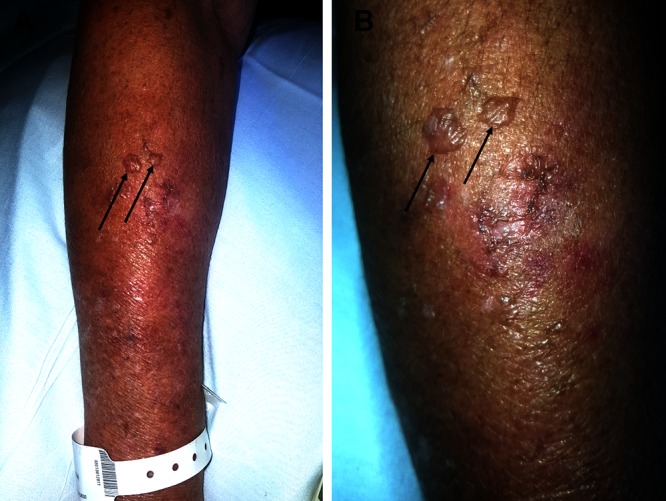
Acute edema bullae mimicking the disseminated herpes zoster. The distant (A) and closer (B) views of clear, individual bullae (arrows) with non-erythematous bases (arrows) on the dorsal right forearm.

After seven days of intravenous antiviral therapy, his VZV-associated lesions had nearly resolved (Figure 1). However, new blisters were noted on the swollen right forearm, proximal to the insertion site of a peripheral venous line that had delivered intravenous acyclovir over the past week. In contrast to his earlier blisters, the new lesions on the right arm were clear without erythema, individual rather than grouped, and non-tender (Figure 2).

**Figure 3:**
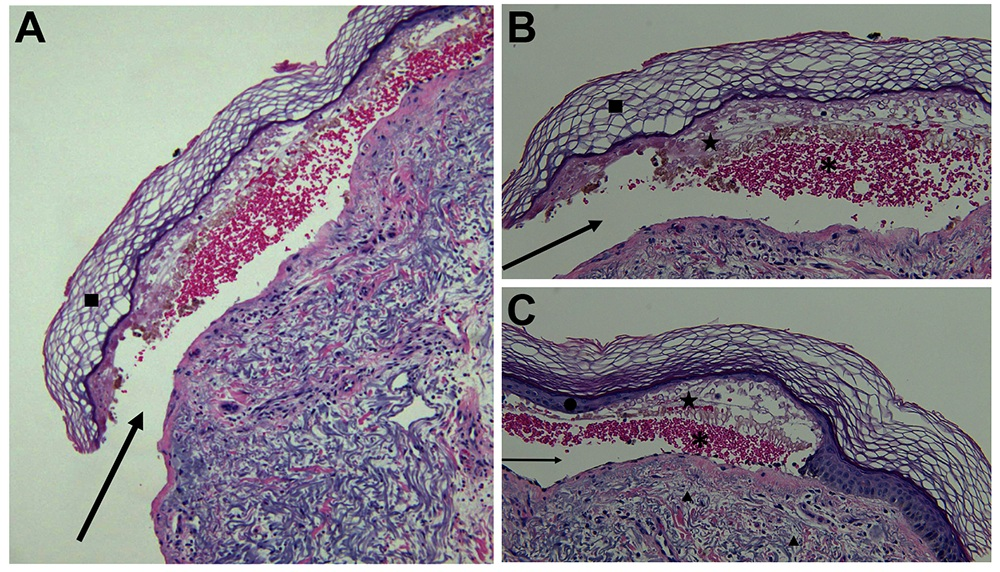
Low (A), intermediate (B), and higher (C) magnification views of the pathologic changes of an edema bulla from a skin biopsy of a blister on the right dorsal forearm of a 53-year-old immunosuppressed male with a prior history of the renal transplant and a recent resolving episode of disseminated varicella zoster virus infection with dermatomal and nondermatomal vesicles. There is a paucicellular subepidermal blister (A). The epidermis is intact overlying the middle portion (B) and lateral edge (C) of the blister. There is orthokeratosis of the stratum corneum (square) and the remainder of the epidermis is thinned with effacement of the rete ridges (circle) forming the roof of the blister. The blister (arrow) is located below the epidermis; it contains serum (star) and red blood cells (asterisk). There is edema, with widening of the spaces between the collagen bundles in the dermis (triangles), inflammatory cells such as lymphocytes are few to absent around the blood vessels and within the surrounding connective tissue (hematoxylin and eosin: A: x4; B: x10 and C: x20).

The polymerase chain reaction testing of the new lesions was negative for varicella zoster virus and herpes simplex virus. Microscopic examination of a biopsy taken from the edge of a blister showed a paucicellular subepidermal vesicle with epithelial necrosis, focal ischemic alteration of the eccrine apparatus, and pronounced dermal edema (Figure 3).

Correlation of the laboratory studies and microscopic examination of the skin biopsy of the new, painless, polymerase chain reaction VZV-negative bullae in a patient with resolving disseminated herpes zoster following one week of antiviral therapy established a diagnosis of acute edema blister. These blisters were likely secondary to lymphedema from intravenous catheter-associated pressure or trauma since the new blisters were located in a limb previously uninvolved with VZV but associated with intravenous catheter access. The peripheral intravenous catheter was relocated to the left limb and lesions resolved.

## Discussion

Edema bullae are the blisters that occur most commonly in the elderly and immobile during acute increases in interstitial fluid pressure [1]. They present as non-erythematous, medium to large-sized bullae with sterile fluid housed under a thin roof that often breaks within a few days [6]. They are sequelae of increased fluid, appearing at both the time and site of edema. As such, the clinical history and physical presentation are typically sufficient for diagnosis; a biopsy showing subepidermal edema with negative immunofluorescence staining may be performed to differentiate acute edema blisters from other bullous diseases such as bullous pemphigoid and pemphigus vulgaris [6].

When capillary filtration develops too rapidly for compensatory lymphatic drainage, dermal edema may occur, leading to blister formation [1]. This occurs most frequently during acute decompensation in hepatic, renal, or cardiac failure. Acute edema blisters may also occur more readily in the patients with poor lymphatic drainage such as those with venous insufficiency or immobility leading to disuse of muscular pumps. Rarely, edema blisters have been reported in hereditary angioedema [6].

The treatment of acute edema blisters is directed towards reducing fluid overload. The elevation of the dependent extremity may promote lymphatic drainage and lesions typically resolve once the fluid imbalance is corrected [1,6]. In our patient, lesions were believed to result from lymphedema from an intravenous catheter, and they resolved once the catheter was transferred to another limb.

The herpes zoster classically manifests as grouped, painful vesicles in a dermatomal distribution. However, disseminated herpes zoster may present with skin lesions simultaneously involving multiple dermatomes (on one or both sides of the body) or with a widespread distribution of more than 20 lesions either alone or be accompanying lesions that are located in one or more dermatomes. The diagnosis is typically established clinically, but confirmation with polymerase chain reaction testing for VZV deoxyribonucleic acid (DNA) or direct fluorescent antibody testing can be performed [3]. Ninety percent of herpes zoster occurs in immunocompetent individuals; however, the risk of developing herpes zoster increases by 20-fold to 100-fold with immunosuppression [7]. Immunosuppressed individuals are also more likely to develop disseminated zoster as well as complications such as visceral, ocular, or neurological involvement [8]. In our immunosuppressed patient, this was the second episode of disseminated herpes zoster.

The treatment of uncomplicated herpes zoster involves oral antiviral therapy for seven to 10 days, while disseminated disease or visceral involvement warrants intravenous therapy. Immunosuppressed patients with uncomplicated herpes zoster may also be treated orally, although intravenous acyclovir is recommended in the severely immunocompromised in order to prevent disseminated disease [3]. This population includes patients who have received stem cell transplants within the preceding four months, the patients with moderate to severe graft-versus-host disease following hematopoietic stem cell transplant, and transplant recipients with high dose immunosuppression [7].

Resolved areas of prior VZV infection may become “immunocompromised districts”, areas of skin with increased susceptibility to other skin disorders, including malignancy (such as cutaneous metastases), opportunistic infections, and granulomatous disorders (such as granuloma annulare and sarcoidosis) [9]. The predisposition for this sequelae is considered to be related to local immune dysregulation, and immunocompromised populations appear to have increased vulnerability especially following VZV infection [9]. However, acute blister formation in our patient did not occur in an area of his previous disseminated VZV infection or a site affected by his currently resolving VZV infection; it was likely secondary to catheter-induced trauma or lymphedema or both.

Many dermatological conditions are known to mimic VZV infection, including cellulitis, contact dermatitis, dermatitis herpetiformis, drug eruptions, herpes simplex infection, and tinea infection [3]. Rarely, zosteriform lesions have also been reported in cutaneous metastases [4], eosinophilic dermatosis of hematologic malignancy [3], and Staphylococcus aureus infection in a dermatomal distribution [2]. In our patient, lesions initially suspected to be continued disseminated herpes zoster infection, were determined by histology and negative polymerase chain reaction testing to be acute edema blisters secondary to skin edema.

## Conclusions

Edema bullae present as the medium to large-sized vesicles and typically manifest during states of volume overload causing acute swelling in the affected site. Our patient’s lesions occurred in the setting immediately following disseminated herpes zoster infection, and were initially misinterpreted as progressive disease. However, the viral studies were negative for VZV and herpes simplex virus, while a lesion biopsy showed a non-inflammatory subepidermal blister; correlation of these studies and the clinical history of recent intravenous therapy near the involved site established the correct diagnosis of acute edema bullae. In conclusion, the acute edema bullae may result from prolonged pressure or trauma from a peripheral venous line; in addition, acute edema bullae can be added to the list of dermatological conditions that may mimic varicella zoster virus infection.
